# Responsive Open Microfluidic Logic for Mechanical Computing

**DOI:** 10.1002/advs.77546

**Published:** 2026-09-01

**Authors:** Roi Almog, Bat‐El Pinchasik

**Affiliations:** ^1^ School of Mechanical Engineering Faculty of Engineering Tel‐Aviv University Tel Aviv Israel; ^2^ Center for Physics and Chemistry of Living Systems Tel Aviv University Tel Aviv Israel

**Keywords:** capillarity, flexural deformations, liquid diodes, logic gates, open microfluidics

## Abstract

Logic circuits, a cornerstone of digital computation, have been increasingly adapted to microfluidic systems to enable autonomous, programmable fluidic operations that can also interact with the environment. Unlike traditional microfluidics, which rely on enclosed channels and external pumps, open microfluidics leverage capillary spontaneous flow to manipulate liquids. Integrating logic into these systems facilitates sequential dispensing, mixing, and reaction triggering, without the need for external electronic inputs or pumps. This study leverages elasto‐capillary coupling to implement logic in open microfluidic platforms, encoding binary operations through geometric constraints and responsive soft materials. We demonstrate the realization of fundamental gates (AND, OR, NOT, and XOR) and more complex circuits with computational capabilities (half‐adder and full‐adder). We construct a motorized actuation system that delivers the mechanical input with high reproducibility, supporting applications in diagnostics, microliter reaction handling, and interaction of fluidic systems with the environment. By combining simplicity in operation with the robustness of logic‐based control, this approach lays the foundation for next‐generation responsive microfluidic systems and mechano‐fluidic computing.

## Graphical abstract

Coupling elasticity with capillarity transforms mechanical compression into binary fluidic states, enabling deterministic logic in open microfluidic circuits. This work demonstrates that computation can emerge from interactions between mechanic and fluidic domains, establishing a general strategy for embedding information processing directly within responsive materials.

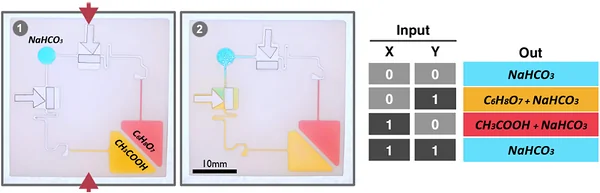

## 1 Introduction

Computation can emerge from diverse physical substrates. Beyond the transport of electrons in silicon, it may be embodied in the motion, deformation, and interaction of matter. In this context, mechanical and fluidic computation represent unconventional paradigms in which logic and arithmetic operations arise from the dynamics of materials themselves.

Liquid and electrical circuits share several analogies, despite operating on different principles and mediums. In microfluidics, liquid analogues of electronic components offer a novel approach for translating computational principles through fluid dynamics [1, 2, 3, 4, 5]. Several efforts have sought to create liquid analogues of electronic components across both macrofluidic and microfluidic regimes, in higher [6, 7], and lower Reynolds numbers, respectively.

In the low Reynolds number regime characteristic of microfluidics, channels can be classified as either closed or open. In closed microfluidics, devices can replicate the behaviors of electronic components such as resistors, capacitors, and transistors, with liquids acting as “current” in place of electricity, and external pumps to provide with the pressure modulation needed for controlling flows within the circuits [3, 7, 8, 9, 10]. For instance, fluidic resistors are created by narrowing channels to increase flow resistance. The Hagen–Poiseuille's law may serve as the liquid analogy to Ohm's law [7], and such attempts yielded 2D circuits with roughly dozen building blocks [4, 11, 12], relying on external pressure to operate [13]. Such circuits usually lack responsiveness to external cues and are preprogrammed to function in a predetermined way [14]. Yet, an example of a responsive closed microfluidic circuit demonstrated a movable bubble used to alternately open and close parts of microfluidic channels, in response to pressure variations within the circuit [15].

In open microfluidic systems, widely used in diagnostics [16] and Lab‐on‐Chip technologies [17], the implementation of logic circuits remains largely undeveloped. This is because flow in open microfluidics relies on capillarity and spontaneous wicking, which cannot be regulated by external pressure. Therefore, realizing logic in these systems demands a new paradigm for introducing externally controlled inputs. In addition, liquid circuits differ fundamentally from electrical circuits in several key aspects. First, liquid wetting is inherently non‐reversible. Specifically, once a liquid propagates through the channels, it cannot readily retract. This imposes irreversibility and introduces a strong dependence on the history and timing of operations [18, 19, 20, 21]. Second, the characteristic time scales of liquid propagation in fluidic circuits are significantly slower, typically on the order of seconds, in contrast to the nanosecond‐scale operation of electronic logic circuits [18, 22, 23, 24, 25]. Finally, the microfluidic domain lacks the concept of an electrical ground, as liquids cannot be instantaneously removed or “reset,” rendering charge‐based grounding concepts inapplicable [26, 27, 28]. Thus, a central challenge remains: how can tunable flow be achieved in spontaneous, pump‐free capillary networks? Addressing this requires the development of a platform composed of responsive building blocks, along with strategies to assemble these units into dynamically reconfigurable networks. Both should rely on wetting mechanisms governed by transient responses to external stimuli rather than by pressure regulation.

Previous microfluidic logic systems have generally relied on pressure‐driven operation in closed channels [29, 30, 31] or on passive capillary networks whose behavior is encoded by fixed channel geometries [4, 32]. In contrast, here we leverage mechanically responsive capillary elements, specifically liquid diodes and capillary switches, to implement logic gates within pump‐free open microfluidic channels. By utilizing the inherent responsiveness of flexible liquid diodes, previously reported as liquid Zener diodes [22], alongside adaptable capillary switches, we establish the positive and negative logic units required for constructing a comprehensive set of logic gates. Building on this foundation, we demonstrate computational capillary circuits capable of performing Boolean arithmetic operations: half‐adder and full‐adder functionalities. Finally, we apply these open fluidic logic circuits to control a bubble‐generating reaction between acetic acid, citric acid, and sodium bicarbonate, exploiting the unique ability of open microfluidics to let generated bubbles escape without obstructing the fluidic pathways. A motorized actuation platform further confirms the robustness and reproducibility of this response, underscoring its potential for small‐volume handling and automized diagnostic applications.

From a broader perspective, this platform offers a means to investigate circuit‐like behaviors in systems where biological fluids, chemicals, or other liquids interact under controlled conditions. Such capabilities hold promise for advancing lab‐on‐a‐chip technologies [33, 34, 35, 36], micro‐reactors [37, 38], developing next‐generation diagnostic tools [34, 39, 40], and enabling liquid‐based mechanical computation [41, 42].

## 2 Results and Discussion

The realization of logic circuits requires the integration of two fundamental functional units. The first is a buffer, which ensures a direct mapping between input and output states (i.e., input 0 yields output 0, input 1 yields output 1). The second is an inverter, which implements logical negation by producing the complement of the input state (i.e., input 0 yields output 1, and input 1 yields output 0). The coexistence of these two complementary elements is essential for enabling complete logical operations and establishing a universal logic framework [43, 44].

**FIGURE 1:**
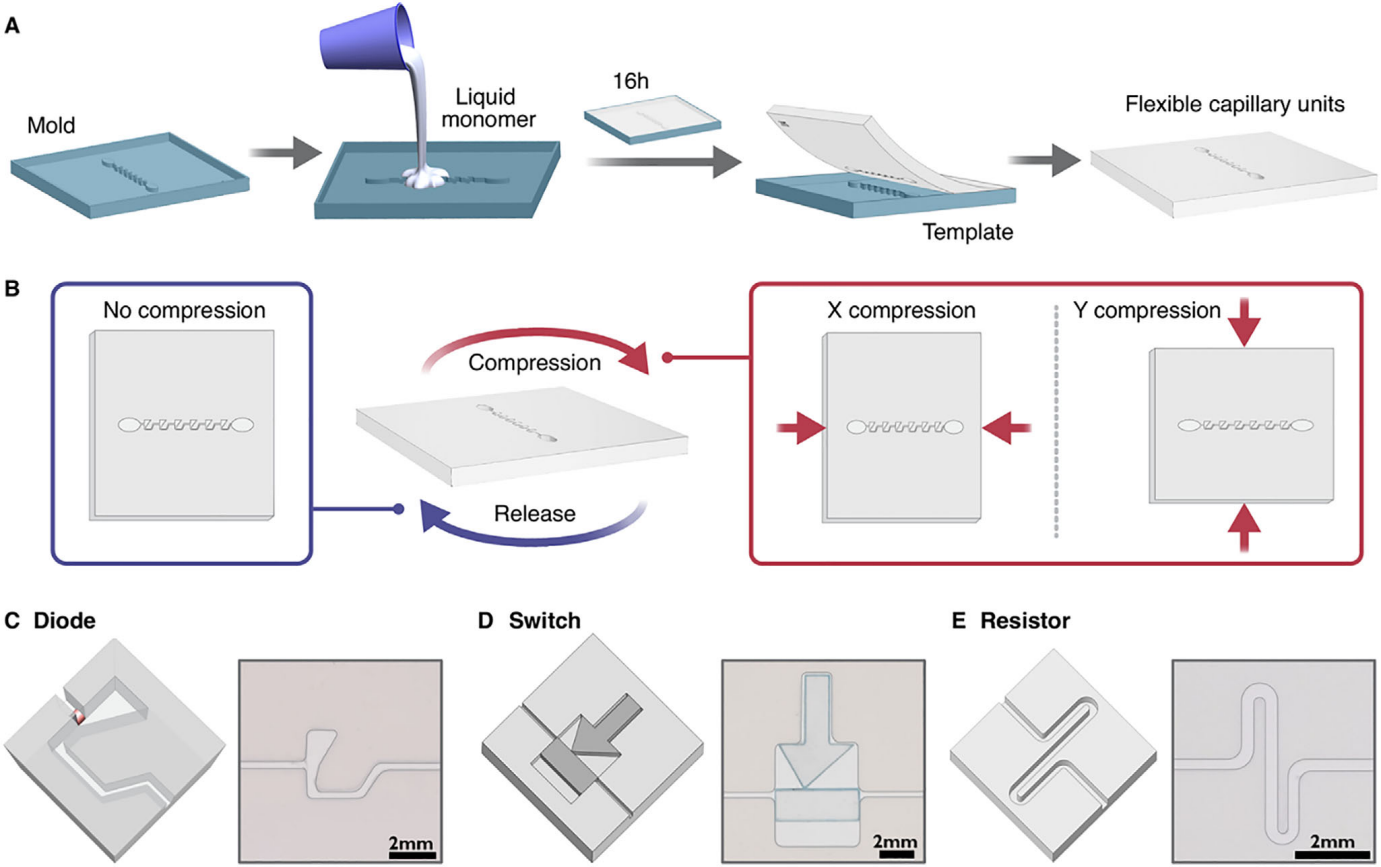
Realization of flexible capillary units for computational open microfluidics. (A) Fabrication process of the flexible capillary units. (B) Activation mechanism of the units: blue indicates the uncompressed state, while red indicates the compressed state, with two distinct compression modes (X and Y). (C–E) Liquid‐analogues of electronic components: Zener diode, switch, and resistor (left to right). For each component, both the CAD model and corresponding optical image are shown.

Implementing capillary logic gates requires elementary stimulus‐responsive units. Figure 1 outlines the fabrication of these flexible components (Figure 1A) and the flexural deformations they are subjected to in this study (Figure 1B). First, a liquid monomer is cast into a 3D‐printed mold, cured, and then released from the template. The resulting flexible open microfluidic module operates in two distinct mechanical states, compressed (red) and released (blue) (Figure 1B). Compression applied along the x‐ and y‐axes is referred to as *x‐compression* and *y‐compression*, respectively.

Figure 1C–E illustrates the three fundamental capillary components forming the basis of the logic gates that enable the responsive computational fluidic circuits demonstrated in this study. For each component, both the computer‐aided design (CAD) model and the corresponding optical image of the molded elastomer are shown: liquid Zener diode (Figure 1C) [22], liquid switch (Figure 1D), and liquid resistor (Figure 1E). The operating principles of these components are detailed in the following sections.

**FIGURE 2:**
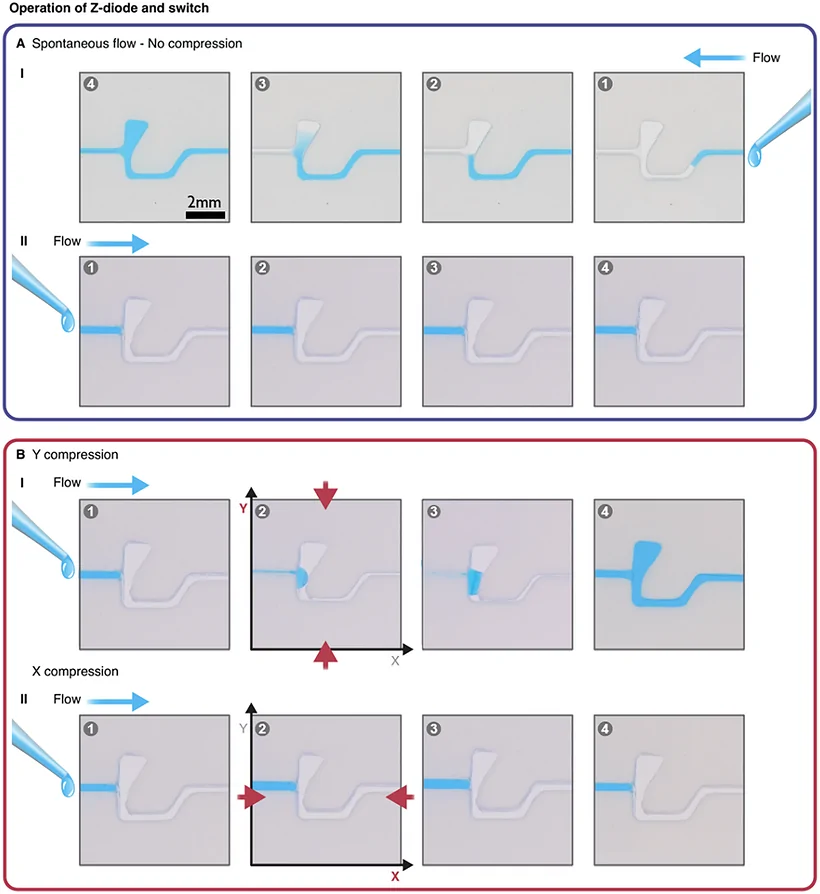
Liquid Zener diode. Optical images of (A) spontaneous flow without compression. (A(I)) Flow in the forward direction. (A(II)) Flow in the backward (blocking) direction. (B) Liquid flow under mechanical actuation. (B(I)) Compression along the Y‐axis induces capillary‐bridge formation, resulting in backflow. (B(II)) Compression along the X‐axis does not induce capillary‐bridge formation; therefore, no fluidic breakdown occurs. The numbering represents temporal chronological order (see Video S1).

Figure 2 presents an optical image of the liquid Zener diode in operation. In the resting, uncompressed, state (blue frame), the device rectifies flow: it permits forward flow (Figure 2A(I)) and suppresses reverse flow (Figure 2A(II)) [22]. In Figure 2A(II), flow halts at the channel outlet due to the abrupt channel expansion. Liquid propagates spontaneously only while the Laplace pressure at the meniscus remains negative. Upon reaching the channel outlet, the contact line pins at the channel edges, causing the meniscus to become nearly flat. As a result, the Laplace pressure approaches zero, eliminating the capillary driving force required for further propagation. To overcome this barrier and break the diodic behavior, a mechanical perturbation compresses the channel along the flow direction (Figure 2B(I)), creating a liquid bulge that contacts the opposing wall and forms a capillary bridge. The newly formed bridge restores a negative Laplace pressure, reestablishing the capillary driving force and allowing the liquid to resume spontaneous propagation [22].

In contrast, when compression is applied parallel to the connecting channel, the tunnel cross‐section remains unchanged. As a result, no sufficient capillary pressure difference is generated to drive liquid protrusion into the tunnel and form the capillary bridge. Consequently, rectification is preserved, and the liquid is halted at the diode entrance (Figure 2B(II)). This anisotropic mechanical response underlies the dependence of liquid position on the history of operations (see Video S1).

**FIGURE 3:**
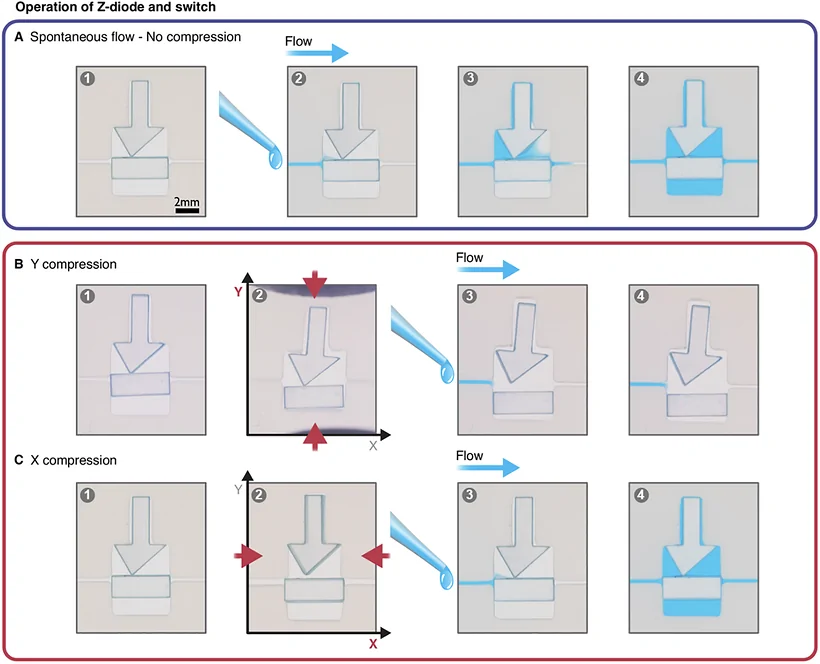
Liquid switch. Optical images of (A) spontaneous flow without compression. (B,C) Liquid propagation under actuation. (B) Compression along the Y‐axis moves the rectangular block, disconnecting the channel. (C) Compression along the X‐axis does not move the block, allowing spontaneous liquid propagation through the unit. The detailed mechanism is shown in Figure S1.

Figure 3 presents optical images of the liquid switch. In the uncompressed state (blue frame), the switch conducts liquids in both directions: a rigid, movable rectangular block spans the gap and creates a capillary flow pathway between the two channels, regardless of the inflow direction (Figure 3A). Under global mechanical compression (red frame), the response depends on the compression direction. When compression is applied perpendicularly to the connecting channel (Figure 3B), the rigid arrow drives the rectangular block away, interrupting the liquid pathway and preventing bridge formation; the switch is thus open (non‐conducting) for either inflow direction. In contrast, when compression is applied parallel to the connecting channel (Figure 3C), the block remains in place, preserving a continuous path; the switch remains closed (conducting). See Video S2 and Figure S1.

**FIGURE 4:**
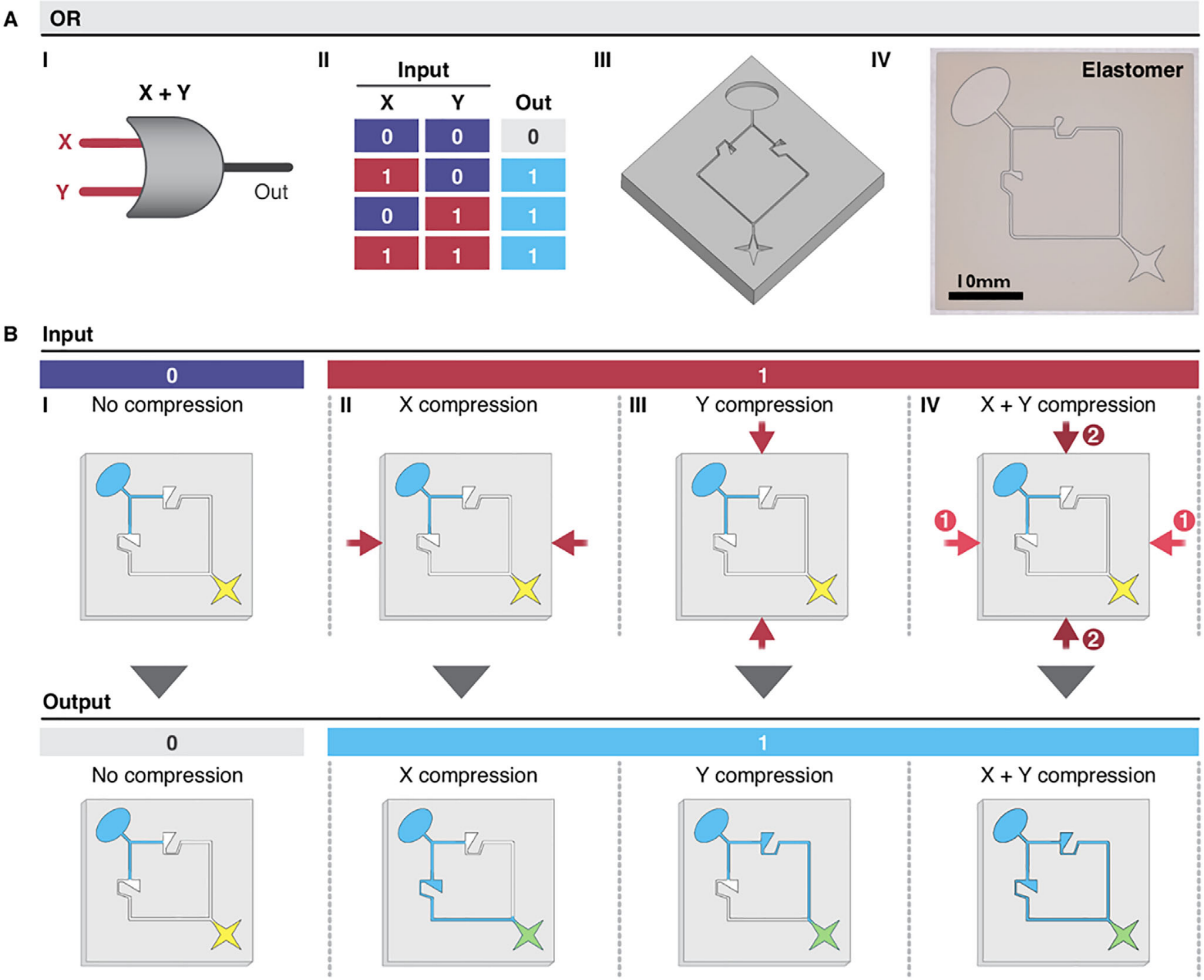
Fluidic OR gate. (A) OR gate representation. (A(I)) schematic symbol with the corresponding logical expression. (A(II)) logic table with color‐coded values (inputs: 0 in blue, 1 in red; outputs: 0 in gray, 1 in azure). (A(III)) CAD model. (A(IV)) Top view of the fabricated flexible capillary circuit. (B) Top row: top‐view images of the four input configurations before their application. Red arrows indicate the direction of applied mechanical compression. Labels “1” and “2” indicate compressions applied sequentially. Bottom row: resulting outputs. Each illustration shows a schematic top view of the final state.

Using the three fundamental fluidic elements, the Zener diode, switch, and resistor, we constructed capillary logic gates by arranging them in specific configurations. The inputs consist of global mechanical actuation, specifically compressions applied along either the X or Y directions, while the output is defined as the liquid reaching the designated endpoint of the circuit. We begin with the basic OR gate (Figure 4A(I)), composed of two liquid Zener diodes positioned in parallel [45].

This configuration defines two independent input signals, physically encoded by mechanical compression along two orthogonal axes, X and Y (Figure 4A(II)). A logical input “0” denotes the absence of compression along the corresponding axis, whereas “1” denotes the application of compression along that axis. Accordingly, the four input states are implemented as 00 = no compression, 10 = compression along X only, 01 = compression along Y only, and 11 = compression along both X and Y. The output is defined separately: “1” corresponds to liquid propagation to the designated output reservoir, whereas “0” indicates that no liquid reaches the corresponding output.

The liquid is introduced into the oval reservoir and advances spontaneously through the open microchannels (liquid contact angle = 53° ± 2°). A CAD model (Figure 4A(III)) and an optical image (Figure 4A(IV)) show the elastomeric microfluidic OR gate. Figure 4B illustrates the operation of the fluidic OR gate under the four possible input conditions: I) no compression, II) X‐compression, III) Y‐compression, and IV) X+Y compression, together with the corresponding fluidic outputs in the circuit (see Video S3).

**FIGURE 5:**
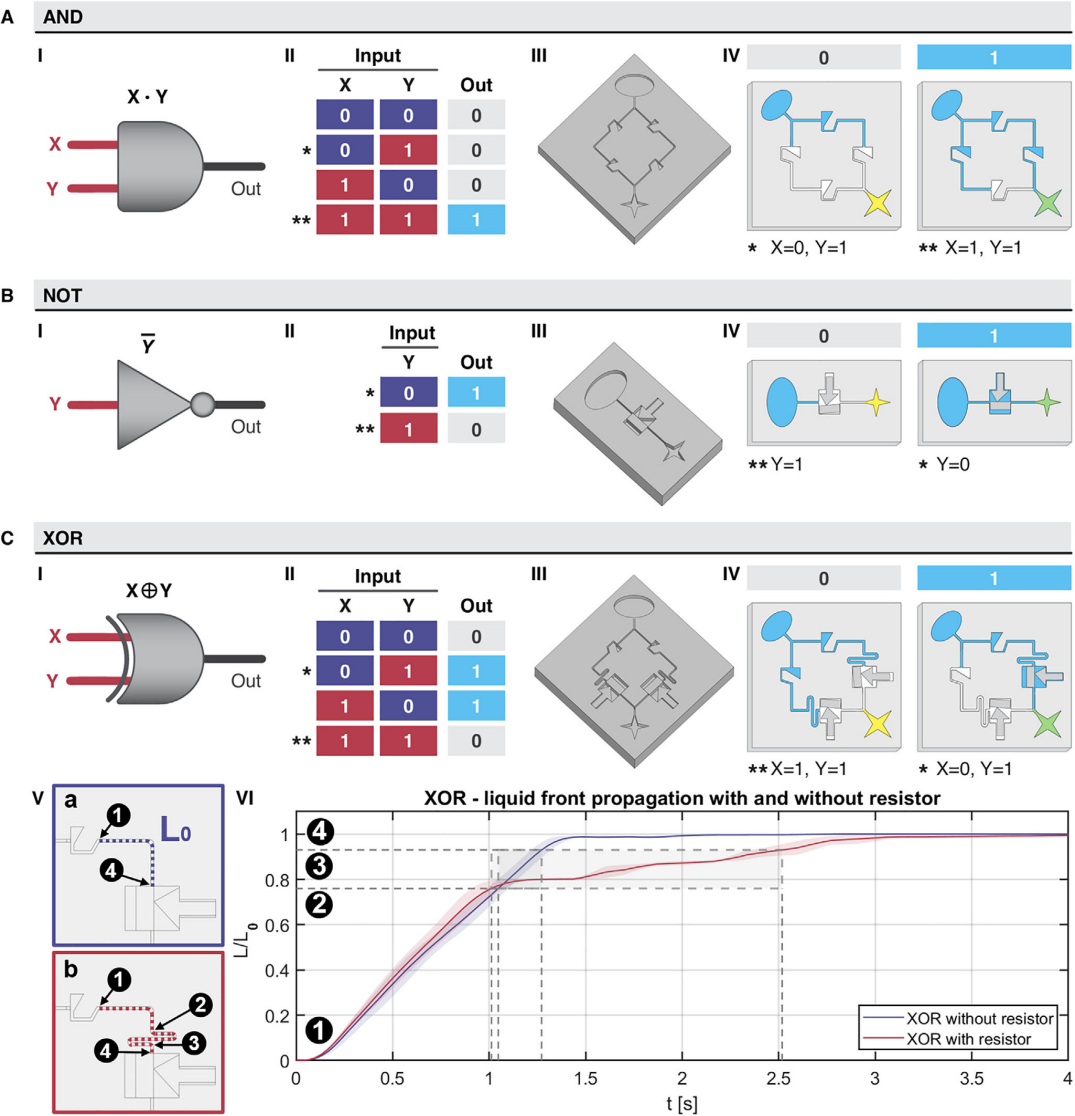
AND, NOT, and XOR gates. (A–C) Representations of the fluidic AND, NOT, and XOR gates. (I) Schematic symbol with the corresponding logical expression. (II) Truth table with color‐coded values (inputs: 0 = blue, 1 = red; outputs: 0 = gray, 1 = azure). (III) CAD model. (IV) Two schematic top views showing the final state of each gate. Each top view corresponds to a specific input, indicated below the illustration. (C(V)) Enlarged top view of the channel between the diode and the switch (a) without and (b) with a resistor. *L*_0_ denotes the end‐to‐end channel length without a resistor. Points 1–4 indicate locations along the channel. (C(VI)) Average liquid‐front propagation in XOR tunnels with and without a resistor. The dashed vertical lines indicate the points at which the liquid enters and exits the resistor. The shaded blue and red regions represent the standard deviation.

Following this logic, we extend the directory of liquid analogues to fluidic AND, NOT, and XOR gates and quantify the operation dynamics (Figure 5). In general, the liquid state within the circuit depends on the sequence of actuations, that is, the order in which compressions along the X and Y directions are applied. To avoid this path‐dependence, we designed the networks such that the output is independent of the actuation order.

Figure 5A(I, II) presents the logic table of the fluidic AND gate, consisting of four Zener diodes arranged as two parallel pairs in series (Figure 5A(III)). Liquid propagation occurs only when compression is applied along both the X‐ and Y‐axes, resulting in an output of “1” (Figure 5A(IV); see Video S4).

Figure 5B(I–IV) shows the fluidic NOT gate, implemented as a single fluidic switch. When compression is applied along Y (input = 1), the liquid flow is halted; without compression, the liquid passes freely through the unit (Video S5). Finally, the fluidic XOR gate (Figure 5C(I–III)) comprises two parallel branches, each containing a Zener diode, a resistor, and a switch, arranged consecutively. A fluidic resistor is incorporated to increase the propagation delay, ensuring that the liquid reaches the switch only after both possible actuation inputs are applied, thereby increasing the available input time window by approximately 1.28 s (Figure 5C(IV)). The logic table (Figure 5C(II)) summarizes its operation: the output is “1” only when compression is applied along exactly one axis (Video S6).

A simple yet fundamental example of useful logic capability is arithmetic, with addition being its most basic operation. In a multi‐digit addition, the first step is to add two digits together; this operation is performed by a half‐adder circuit. However, as its name suggests, the half‐adder can perform only part of the operation: it cannot process a *Carry* digit. The *Carry* becomes essential when longer additions are required. A full‐adder circuit incorporates this third input, enabling the combination of three digits, two operands and a *Carry*, into a single summed output.

**FIGURE 6:**
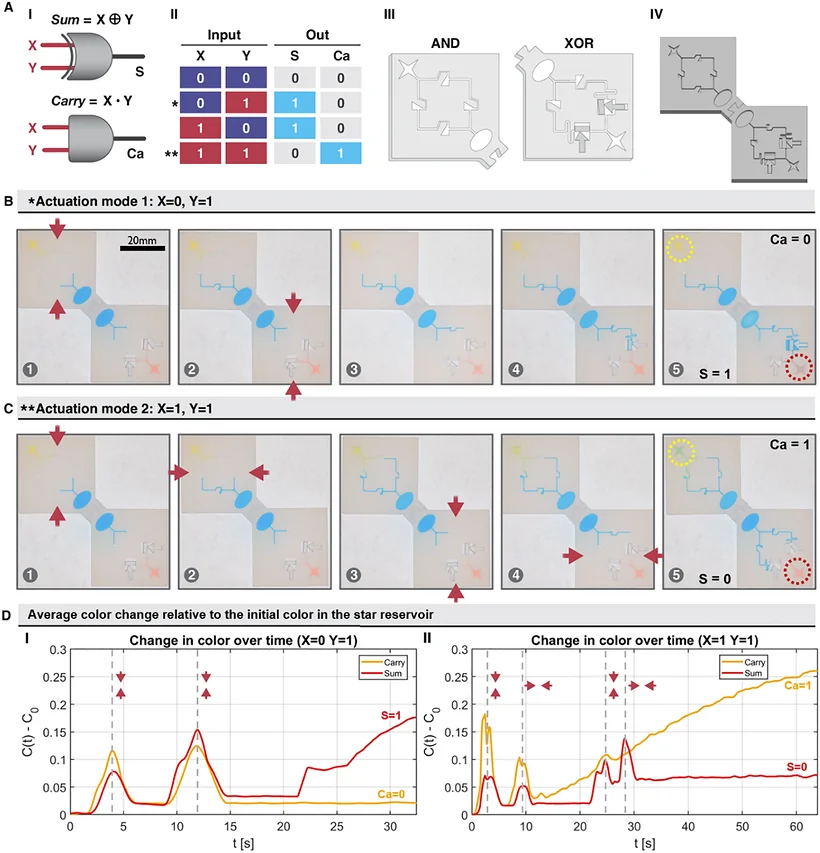
Fluidic half‐adder circuit. (A) (A(I)) Schematic symbols and logical expressions of the AND gate (*Carry*) and XOR gate (*Sum*). (A(II)) Truth table with color‐coded inputs (0 = blue, 1 = red) and outputs (0 = gray, 1 = azure). (A(III)) Schematic top views of each fluidic gate, and (A(IV)) the corresponding CAD models showing their interconnection via a puzzle‐like joint. Optical images showing (B) operation for inputs X = 0 and Y = 1, with red arrows indicating the direction of compression, and (C) operation for inputs X = 1 and Y = 1. The final readouts are indicated by yellow and red dashed circles for the *Sum* and *Carry* circuits, respectively. (D) Time‐dependent color changes, *C*(*t*), at the readout endpoints, relatively to the initial color *C*_0_. *Carry* is shown in yellow and *Sum* in red. Half‐adder *Carry* and *Sum* signals over time for X = 0 and Y = 1 (D(I)) and X = 1 and Y = 1 inputs (D(II)). The dashed gray lines indicate the timing of the compressions, and the red arrows indicate the direction of the corresponding compression. Detailed time series of the full operation is shown in Figures S3 and S4.

Having established the basic arithmetic logic of half‐ and full‐adders, we next implement these operations in our capillary platform. Figure 6 presents the operation of a fluidic half‐adder circuit that executes fundamental binary arithmetic, producing both *Sum* (also denoted S) and *Carry* (also denoted Ca) outputs. The *Sum* represents the least significant bit and the *Carry* the most significant bit of the computation. In our implementation, the *Sum* operation corresponds to XOR, and the *Carry* corresponds to AND. We apply the inputs to both the *Sum* and *Carry* circuits separately according to the schematic symbols (Figure 6A).

Figure 6B presents an optical image sequence of the half‐adder circuit computing 0 + 1. In this operation, the XOR gate generates the *Sum* output (red liquid in the star reservoir), while the AND gate produces the *Carry* output (yellow liquid in the star reservoir). The *Carry* is shown to form first, followed by the *Sum*; however, the final readout is independent of the order of actuation. The outputs are *Sum* = 1 and *Carry* = 0, corresponding to the binary outcome 01 (decimal 1) (see Video S7).

Figure 6C presents an optical image sequence of the half‐adder circuit computing 1 + 1. As in the previous case, the *Carry* was evaluated first, followed by the *Sum*. The resulting outputs are *Sum* = 0 and *Carry* = 1, corresponding to the binary result 10 (see Video S7). The normalized color intensity in the star reservoir was tracked over time (Figure 6D) and compared to the initial intensity, enabling continuous monitoring of the binary *Carry* and *Sum* outputs.

**FIGURE 7:**
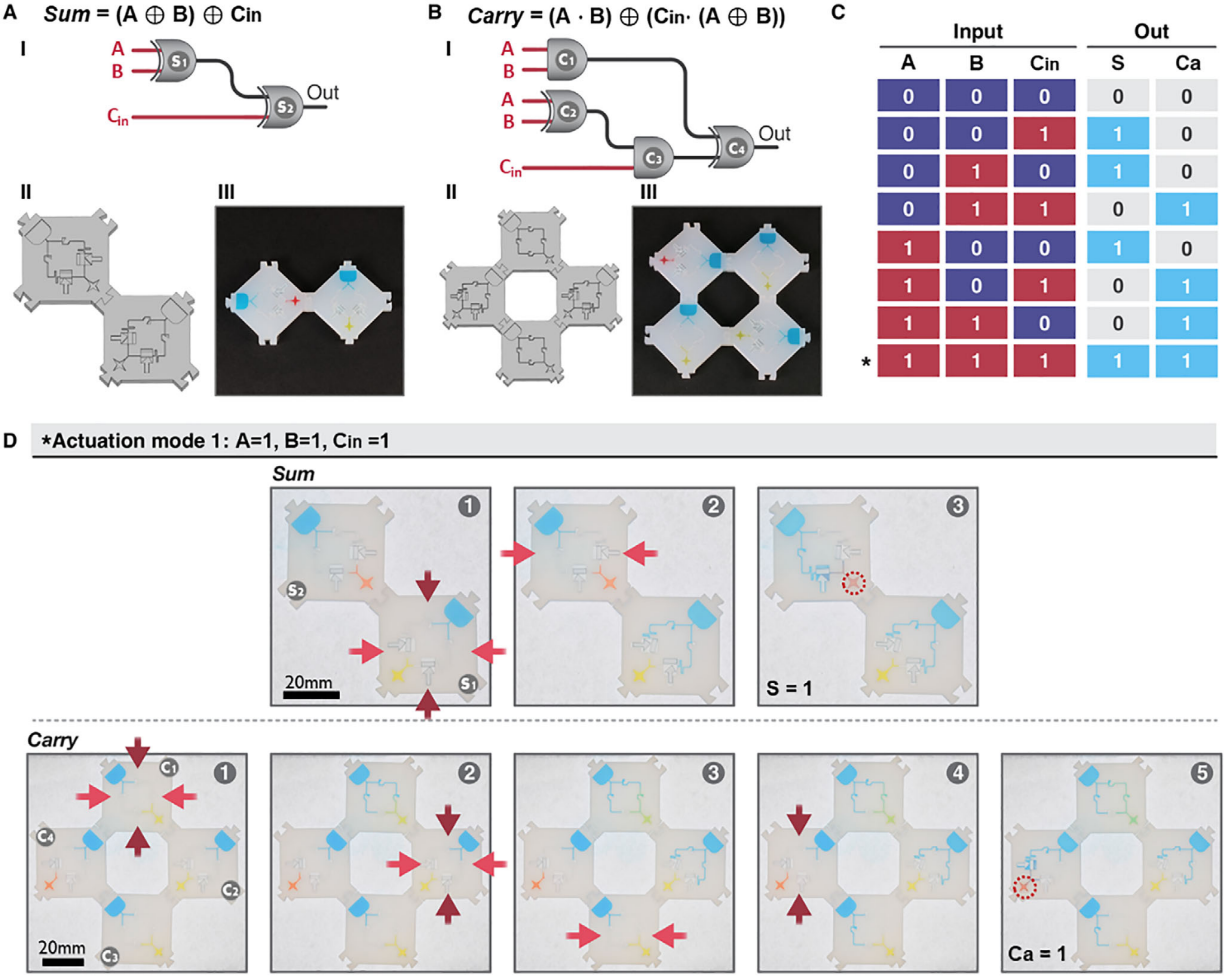
Fluidic full‐adder circuit. (A,B) (I) Logic diagram with the corresponding mathematical expression for the *Sum* (A), constructed from two XOR gates, and *Carry* (B) constructed from two AND gates and two XOR gates. (II) CAD models, in which the gates are interconnected through a puzzle‐like joint. (III) Isometric view of the silicone model. (C) Truth table with color‐coded values (inputs: 0 in blue, 1 in red; outputs: 0 in gray, 1 in azure). Three inputs (A, B, and C_in_) and two outputs (S for *Sum* and Ca for *Carry*) are defined. (D) Sequential optical images showing operation with A = 1, B = 1, and C_in_ = 1 inputs. Dark red arrows indicate Y‐compression and light red arrows indicate X‐compression. Note that the compressions are applied sequentially (detailed time series of the full operation is shown in Figures S5–S7). The final readouts are indicated by red dashed circles for both the *Sum* and *Carry* circuits.

To expand the system's computational capacity, we developed modular, interlocking (“puzzle‐like”) gates that form a reconfigurable architecture. This design enables intuitive, mix‐and‐match assembly of fluidic circuits tailored to specific logical operations. Using these modules, we constructed a full‐adder circuit (Figure 7) capable of performing three‐input binary addition (1 + 1 + 1). The logical inputs are denoted as A, B, and C_in_ to reflect the sequential architecture of the circuit, in which the output of one logic gate serves as the input to the next. Thus, additional logical inputs are introduced by cascading two‐input gates, rather than by requiring additional global compression directions. Each gate therefore operates using its own local X‐Y actuation axes.

Figure 7D demonstrates the computation of 1+1+1, corresponding to the binary result 11 (see Video S8). The outputs of specific gates act as inputs for subsequent ones, producing a sequential series of operations, a hallmark of multi‐level fluidic logic. Specifically, the liquid is introduced before any compression, such that all reservoirs are initially filled. In these circuits, yellow‐star reservoirs represent intermediate gates, while red‐star reservoirs denote final outputs. For the *Sum* function two XOR gates are used. The first receives inputs A = 1 and B = 1, producing A⊕B = 0, which is then combined with input C_in_ = 1 at the second XOR gate. In Figure 7D, images show the actuation of the first gate (label “S1”) followed by the actuation of the second gate (label “S2”). Resulting in output of *Sum* = 1. The *Carry* circuit is derived using the same procedure and rules as the sum circuit, producing an output of *Carry* = 1. Together, these results demonstrate that open microfluidic logic circuits can reliably process information across multiple interconnected capillary units while maintaining autonomous, pump‐free operation.

**FIGURE 8:**
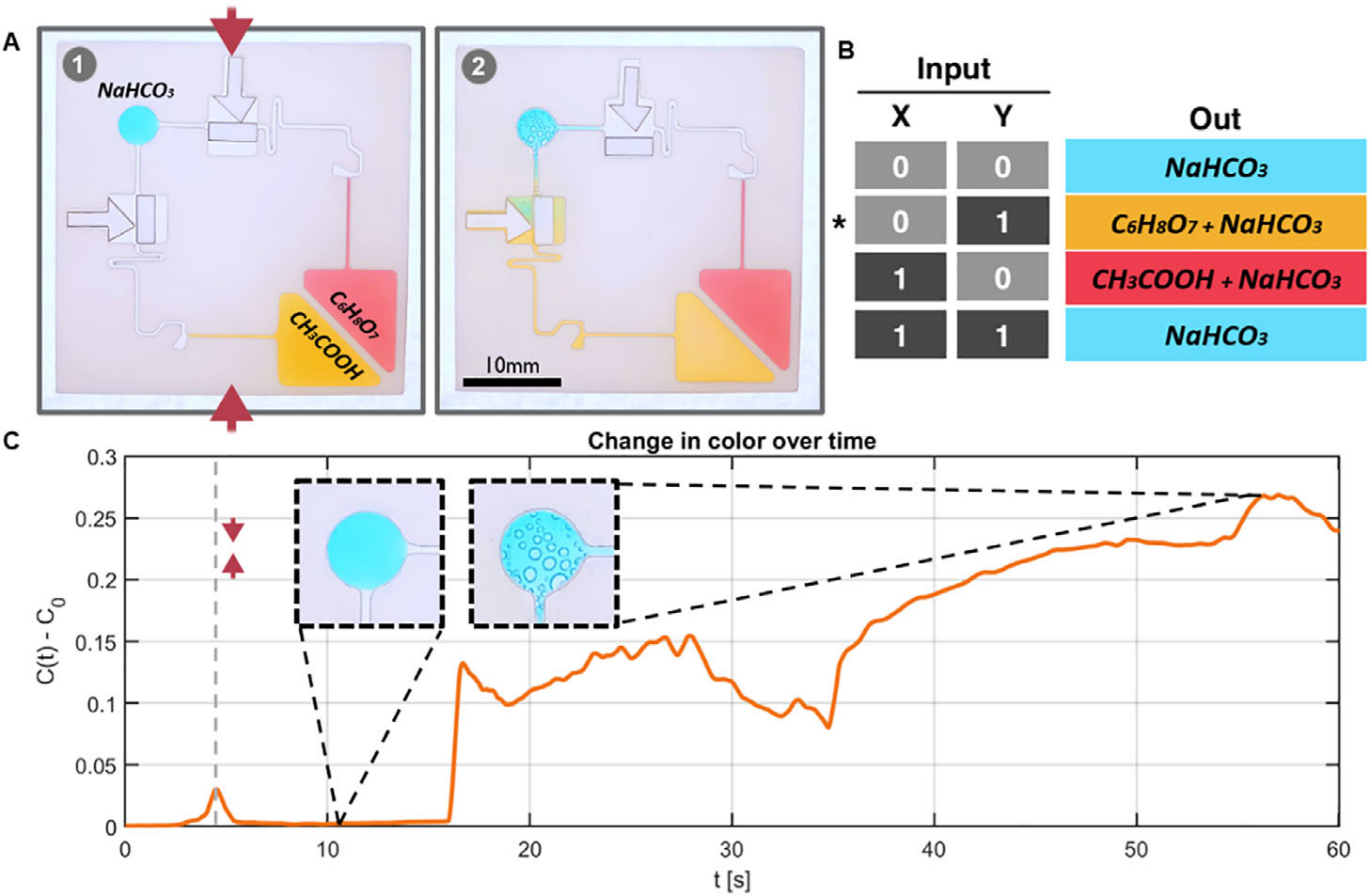
Controlled reaction of acetic acid and citric acid with sodium bicarbonate in an XOR gate. (A) Still images before (1) and after (2) actuation. (B) Logic table showing the corresponding reaction outputs. (C) Average color change relative to the initial color in the final reservoir. The inset images, outlined by black dashed borders, correspond to the indicated time points and highlight the color change and bubble formation in the final reservoir before and after the reaction. Inset figures illustrate the formation of carbon dioxide gas bubbles as a byproduct of the reaction.

Figure 8 demonstrates a potential application of the presented logic gates for programmable reaction selection. The circuit uses fluidic logic to control the spatiotemporal distribution of reagents, selectively routing either acetic acid or citric acid toward sodium bicarbonate according to the applied mechanical input. For the input condition shown here, the acetic acid‐sodium bicarbonate reaction is selected, producing gas bubbles in the final reservoir and confirming successful reaction (Figure 8A (2)). This functionality is uniquely enabled by open microfluidics, where the generated bubbles escape without interfering with circuit operation [46, 47, 48], allowing logic‐controlled bubble‐generating.

**FIGURE 9:**
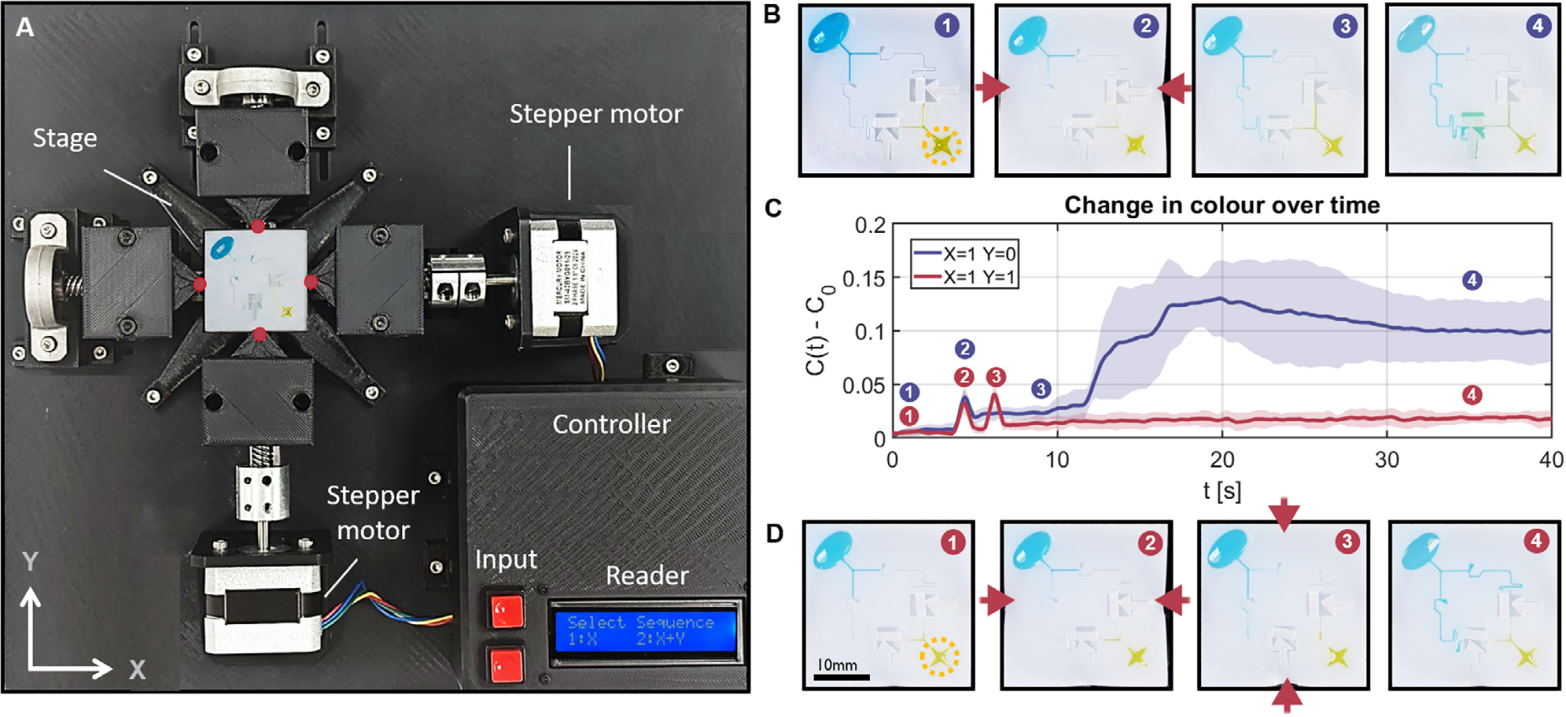
Automated compression system demonstrating XOR‐gate operation. (A) System components, with white labels indicating the local X and Y actuation axes. (B) Still‐image sequence for input state (X = 1, Y = 0). (C) Average color change over time within the region of interest (yellow dashed lines, panels B1 and D1); numbered markers correspond to still images (blue: panel B, red: panel D), with shaded regions showing standard deviation. (D) Still‐image sequence for input state (X = 1, Y = 1); red arrows indicate compression along the corresponding axis.

Finely, to further validate the practical operability of the mechanoresponsive color‐changing system logic system, we implemented an automated compression platform to actuate the XOR gate (Figure 9). This system enables precise, independent control over the two local actuation axes (X and Y), allowing programmable delivery of binary inputs and real‐time readout of the corresponding logical output (See Figure S8 for a detailed description of the system, and Video S10 for the system operation). As shown for the input states (X = 1, Y = 0) and (X = 1, Y = 1), the gate exhibits distinct, reproducible color‐change responses that faithfully track the underlying XOR logic (Figure 9B–D). Quantitative analysis of the average color change within the region of interest confirms the reliability and consistency of the response across repeated actuation cycles. These results demonstrate that the mechanochromic XOR gate can be seamlessly integrated with automated actuation systems, highlighting its potential for scalable, machine‐driven logic operations in soft material‐based computing platforms.

## 3 Conclusions

This work presents a pioneering integration of four key aspects in microfluidics: deformable and responsive networks subject to global mechanical actuation; open channels that interact with the environment; spontaneous flow independent of external pumps; and the ability to implement computational capabilities within fluidic circuits.

We introduce novel mechanically responsive hydrodynamic microfluidic circuits that compute through the interplay of elasticity and capillarity, actuated in situ by external mechanical cues rather than preprogrammed control. Built on open capillary microchannels, this platform performs on‐chip computation, logic, and fluidic signal processing through direction‐selective actuated flow. Using three modular basic units, a liquid Zener diode, a mechanically gated switch, and a fluidic resistor, we compose logic gates and conditioning elements that transform liquid‐borne inputs into deterministic Boolean outputs. The architecture leverages passive capillarity, exploits interactions between liquid and deformable structures, and can be triggered on demand to reshape the meniscus and route the liquid. Collectively, these capabilities advance hydrodynamic circuit design and established a reconfigurable framework for liquid‐based mechanical computing.

Beyond logic circuits, computational fluidics opens a path to explore morphing networks, in which the channels are soft and reconfigurable, thus, their topology can be altered by mechanical actuation. Information can be encoded in non‐volatile capillary states (pinned menisci, trapped droplets, phase partitions). In such networks, both the architecture and liquid output become programmable: routes, junction priorities, and effective resistances can be time‐multiplexed to introduce sequence‐dependent flow and a notion of fluidic memory. This reconfigurability enables discussion of data storage, including the amount of data that can be stored in capillary networks, both in model biological systems and in engineered circuits. From an engineering perspective, such stimulus‐responsive actuation strategies are particularly valuable for assays requiring minimal infrastructure, including point‐of‐care diagnostics, organ‐on‐chip devices, and cell‐based screening systems. By eliminating the need for conventional pressure‐driven control, open microfluidic actuation offers a robust and low‐power mechanism for integrating complex workflows directly into compact, accessible bioengineering platforms. Finally, we demonstrate that open channels enable reactions that generate gas without risking channel blockage. This direct interaction with the surrounding environment also offers potential for sensing applications, such as the detection of environmental contaminants.

While the responsive logic units demonstrated here enable computational fluidics, several challenges remain. A native XNOR gate, has not yet been realized, as its direct implementation would require reliable, simultaneous actuation of three components; for now, XNOR is emulated by combining XOR and NOT. Scaling the actuation scheme beyond two parallel elements reduces reliability, particularly when mixing different logic units, and timing further limits circuit complexity: although front‐end Zener diodes help standardize triggering for AND/XOR gates, NOT operations require tighter control of liquid‐front propagation. To address this issue, the automated actuation system introduced in Figure 9 reduces variability associated with manual actuation by applying the mechanical inputs with controlled and repeatable timing, sequence, and displacement. The lack of a grounding mechanism also constrains inverter design; unlike electronic systems, capillary flows cannot be reset by external forces such as gravity, prompting the development of a liquid switch that locally modulates channel dimensions to achieve inversion. This switch additionally acts as a timer, if the liquid front reaches it before actuation, it reports a fast upstream flow (e.g., from a high‐concentration biomarker), enabling NOT‐based detection. Similar logic‐based preprocessing has been demonstrated previously, but here the gates operate within open microfluidic channels, retaining logical complexity while enabling direct interaction with the environment.

In a broader perspective, we demonstrate that logical behavior can arise when two distinct physical domains are coupled, capillarity and elasticity, producing nonlinear and threshold‐like responses unattainable by either field alone. In our system, the coupling between mechanical compression with capillary flow allows deformation to modulate the Laplace pressure in the liquid, transforming a continuous input into a binary flow state. Such interactions illustrate how cross‐domain coupling, whether mechanical‐capillary, electro‐chemical, or thermo‐elastic, can yield deterministic logic without electronics, providing a general framework for computation embedded directly in physical materials.

## 4 Methods

### 4.1 Sample Design and Preparation

Molds were designed using the CAD software SOLIDWORKS (2024). Each mold consisted of channels comprising 4 unit cells and 2 reservoirs on each side. Pitch heights of 20% were used for the liquid Zener diodes. Once the molds were prepared, the silicone elastomer DRAGONSKIN 30 from Smooth‐On (Macungie, Pennsylvania, United States) was poured into them. This material ensures full polymerisation on the 3D‐printed molds and a shore hardness of 30A, which provides the samples with a good degree of stretchability as well as structural stability. Part A and part B of the elastomer were mixed in equal parts, mixed and then degassed in a desiccator to remove all air bubbles. The mixture was then let cure for 16 h at ambient temperature. After curing, the samples were easily detached from the molds.

The liquid switch's parts were 3D printed using the ProJet MJP 2500 from 3D Systems (Rock Hill, South Carolina, USA). After printing, the parts were cleaned from the support wax. First, a heated oil bath (60°C) was used to dissolve the wax. Then, a heated soapy water bath (soap‐to‐water volume ratio 10%) 60°C was used to remove the oil residues. The parts were lastly rinsed with IPA to ensure removal of all soap residues, and dried with an airgun.

### 4.2 Flow Experiments

Water was mixed with isopropyl alcohol (IPA) at a 5:3 volumetric ratio, resulting in a contact angle of 53° ± 3° on the elastomer. A total of 150 mL of the mixture, colored with blue food dye, was placed in the elliptical reservoir. Distilled water colored with either red or yellow food dye was placed in the star‐shaped reservoir. Food dye was added to all liquids to improve visualization.

### 4.3 Force Measurement

Force measurements were performed using a Mechanical Testing Bench (TA Instruments, USA).

### 4.4 Imaging

The samples were placed on a LED lamp surface for getting better photos and videos. Photos and videos were captured using a Panasonic DC‐S1 camera equipped with a Sigma 70 mm f/2.8 DG Macro Art lens (Sigma Corporation, Japan), providing a 1:1 magnification ratio for high‐resolution close‐up imaging.

The videos were processed using custom MATLAB image‐processing scripts from the raw video files. For the color analysis, regions of interest were manually selected, and the mean RGB vector was calculated for each frame. The color‐change signal was defined as the change relative to the initial mean RGB vector and was smoothed using a moving‐window averaging procedure to reduce noise and transient spikes. For liquid‐front propagation analysis, the tunnel path was manually defined, and the position of the liquid front along this path was tracked over time.

## Author Contributions

**Roi Almog**: conceptualization, investigation, writing – original draft, methodology, validation, visualization, writing – review and editing, software, formal analysis. **Bat‐El Pinchasik**: conceptualization, funding acquisition, writing – original draft, methodology, writing – review and editing, formal analysis, supervision, resources.

## Funding

This research was supported by the Israel Science Foundation (grant No. 822/24).

## Conflicts of Interest

The authors declare no conflicts of interest.

## Supporting information




**Supporting File 1**: advs77546‐sup‐0001‐SuppMat.docx.

**Supporting File 2**: advs77546‐sup‐0002‐VideoS1.mov.

**Supporting File 3**: advs77546‐sup‐0003‐VideoS2.mov.

**Supporting File 4**: advs77546‐sup‐0004‐VideoS3.mov.

**Supporting File 5**: advs77546‐sup‐0005‐VideoS4.mov.

**Supporting File 6**: advs77546‐sup‐0006‐VideoS5.mov.

**Supporting File 7**: advs77546‐sup‐0007‐VideoS6.mov.

**Supporting File 8**: advs77546‐sup‐0008‐VideoS7.mov.

**Supporting File 9**: advs77546‐sup‐0009‐VideoS8.mov.

**Supporting File 10**: advs77546‐sup‐0010‐VideoS9.mov.

**Supporting File 11**: advs77546‐sup‐0011‐VideoS10.mov.

## Data Availability

The data that support the findings of this study are available from the corresponding author upon reasonable request.

## References

[1] A. O. Olanrewaju , A. Robillard , M. Dagher , and D. Juncker , “Autonomous Microfluidic Capillaric Circuits Replicated From 3D‐Printed Molds,” Lab on a Chip 16, no. 19 (2016): 3804–3814, 10.1039/C6LC00764C. 27722504 PMC5314688

[2] M. Yafia , O. Ymbern , A. O. Olanrewaju , et al., “Microfluidic Chain Reaction of Structurally Programmed Capillary Flow Events,” Nature 605, no. 7910 (2022): 464–469, 10.1038/s41586-022-04683-4. 35585345

[3] R. Safavieh and D. Juncker , “Capillarics: Pre‐Programmed, Self‐Powered Microfluidic Circuits Built From Capillary Elements,” Lab on a Chip 13, no. 21 (2013): 4180–4189, 10.1039/C3LC50691F. 23978958

[4] A. Olanrewaju , M. Beaugrand , M. Yafia , and D. Juncker , “Capillary Microfluidics in Microchannels: From Microfluidic Networks to Capillaric Circuits,” Lab on a Chip 18, no. 16 (2018): 2323–2347, 10.1039/C8LC00458G. 30010168

[5] Q. Zhang , M. Zhang , L. Djeghlaf , et al., “Logic Digital Fluidic in Miniaturized Functional Devices: Perspective to the Next Generation of Microfluidic Lab‐on‐Chips,” Electrophoresis 38, no. 7 (2017): 953–976, 10.1002/elps.201600429. 28059451

[6] J. M. Kirshner , Design Theory of Fluidic Components (Academic Press, 2012).

[7] Z. Li , C. Liu , and J. Sun , “Hydraulic–Electric Analogy for Design and Operation of Microfluidic Systems,” Lab on a Chip 23, no. 15 (2023): 3311–3327, 10.1039/D3LC00265A. 37427584

[8] K. W. Oh , K. Lee , B. Ahn , and E. P. Furlani , “Design of Pressure‐Driven Microfluidic Networks Using Electric Circuit Analogy,” Lab on A Chip 12, no. 3 (2012): 515–545, 10.1039/C2LC20799K. 22179505

[9] A. A. Stanley , E. S. Roby , and S. J. Keller , “High‐Speed Fluidic Processing Circuits for Dynamic Control of Haptic and Robotic Systems,” Science Advances 10, no. 14 (2024): adl3014, 10.1126/sciadv.adl3014. PMC10990265 38569043

[10] B. Mosadegh , C.‐H. Kuo , Y.‐C. Tung , et al., “Integrated Elastomeric Components for Autonomous Regulation of Sequential and Oscillatory Flow Switching in Microfluidic Devices,” Nature Physics 6, no. 6 (2010): 433–437, 10.1038/nphys1637. 20526435 PMC2880544

[11] E. Fu , S. A. Ramsey , P. Kauffman , B. Lutz , and P. Yager , “Transport in Two‐Dimensional Paper Networks,” Microfluidics and Nanofluidics 10, no. 1 (2011): 29–35, 10.1007/s10404-010-0643-y. 22140373 PMC3228841

[12] H. Song , Y. Wang , and K. Pant , “System‐Level Simulation of Liquid Filling in Microfluidic Chips,” Biomicrofluidics 5, no. 2 (2011): 024107, 10.1063/1.3589843. 21673845 PMC3112184

[13] L. Li , J. Mo , and Z. Li , “Nanofluidic Diode for Simple Fluids Without Moving Parts,” Physical Review Letters 115, no. 13 (2015): 134503, 10.1103/PhysRevLett.115.134503. 26451560

[14] L. F. Cheow , L. Yobas , and D.‐L. Kwong , “Digital Microfluidics: Droplet Based Logic Gates,” Applied Physics Letters 90, no. 5 (2007): 054107, 10.1063/1.2435607.

[15] M. Prakash and N. Gershenfeld , “Microfluidic Bubble Logic,” Science 315, no. 5813 (2007): 832–835, 10.1126/science.1136907. 17289994

[16] Q. Zhang , S. Feng , L. Lin , S. Mao , and J.‐M. Lin , “Emerging Open Microfluidics for Cell Manipulation,” Chemical Society Reviews 50, no. 9 (2021): 5333–5348, 10.1039/D0CS01516D. 33972984

[17] J. Berthier and K. A. Brakke , “Open Microfluidics,” The Physics of Microdroplets (John Wiley & Sons, 2012), 209–250, 10.1002/9781118401323.ch8.

[18] C. Sammartino , Y. Shokef , and B.‐E. Pinchasik , “Percolation in Networks of Liquid Diodes,” The Journal of Physical Chemistry Letters 14, no. 34 (2023): 7697–7702, 10.1021/acs.jpclett.3c01885. 37606508 PMC10476187

[19] A. M. J. Edwards , R. Ledesma‐Aguilar , M. I. Newton , C. V. Brown , and G. McHale , “Not Spreading in Reverse: The Dewetting of a Liquid Film Into a Single Drop,” Science Advances 2, no. 9 (2016): 1600183, 10.1126/sciadv.1600183. PMC5040479 27704042

[20] A. P. Singh , M. Tintelott , E. Moussavi , et al., “Logic Operations in Fluidics as Foundation for Embedded Biohybrid Computation,” Device 1, no. 6 (2023): 100220, 10.1016/j.device.2023.100220.

[21] G. Kumar and K. N. Prabhu , “Review of Non‐Reactive and Reactive Wetting of Liquids on Surfaces,” Advances in Colloid and Interface Science 133, no. 2 (2007): 61–89, 10.1016/j.cis.2007.04.009. 17560842

[22] C. Sammartino and B.‐E. Pinchasik , “Liquid Zener Diodes,” Materials Horizons 11, no. 20 (2024): 4925–4931, 10.1039/D4MH00688G. 39324522

[23] C. Sammartino , M. Rennick , H. Kusumaatmaja , and B.‐E. Pinchasik , “Three‐Dimensional Printed Liquid Diodes With Tunable Velocity: Design Guidelines and Applications for Liquid Collection and Transport,” Physics of Fluids 34, no. 11 (2022): 112113, 10.1063/5.0122281.

[24] E. W. Washburn , “The Dynamics of Capillary Flow,” Physical Review 17, no. 3 (1921): 273–283, 10.1103/PhysRev.17.273.

[25] H. Kusumaatmaja , “Capillary Filling in Patterned Channels,” Physical Review E 77, no. 6 (2008): 77, 10.1103/PhysRevE.77.067301. 18643402

[26] S. Wang , X. Zhang , C. Ma , S. Yan , D. Inglis , and S. Feng , “A Review of Capillary Pressure Control Valves in Microfluidics,” Biosensors 11, no. 10 (2013): 405, 10.3390/bios11100405. PMC8533935 34677361

[27] H. B. Eral , D. J. C. M. 't Mannetje , and J. M. Oh , “Contact Angle Hysteresis: A Review of Fundamentals and Applications,” Colloid and Polymer Science 291, no. 2 (2025): 247–260, 10.1007/s00396-012-2796-6.

[28] K.‐Y. Law , “Contact Angle Hysteresis on Smooth/Flat and Rough Surfaces. Interpretation, Mechanism, and Origin,” Accounts of Materials Research 3, no. 1 (2022): 1–7, 10.1021/accountsmr.1c00051.

[29] J. A. Weaver , J. Melin , D. Stark , S. R. Quake , and M. A. Horowitz , “Static Control Logic for Microfluidic Devices Using Pressure‐Gain Valves,” Nature Physics 6, no. 3 (2010): 218–223, 10.1038/nphys1513.

[30] M. Rhee and M. A. Burns , “Microfluidic Pneumatic Logic Circuits and Digital Pneumatic Microprocessors for Integrated Microfluidic Systems,” Lab on a Chip 9, no. 21 (2009): 3131–3143, 10.1039/b904354c. 19823730 PMC2917228

[31] N. S. G. K. Devaraju and M. A. Unger , “Pressure Driven Digital Logic in PDMS Based Microfluidic Devices Fabricated by Multilayer Soft Lithography,” Lab on a Chip 12, no. 22 (2012): 4809, 10.1039/c2lc21155f. 23000861

[32] D. Zaremba , S. Błoński , and P. M. Korczyk , “Integration of Capillary–Hydrodynamic Logic Circuitries for Built‐In Control Over Multiple Droplets in Microfluidic Networks,” Lab on a Chip 21, no. 9 (2021): 1771–1778, 10.1039/D0LC00900H. 33710202

[33] P. Neužil , S. Giselbrecht , K. Länge , T. J. Huang , and A. Manz , “Revisiting Lab‐on‐a‐Chip Technology for Drug Discovery,” Nature Reviews Drug Discovery 11, no. 8 (2012): 620–632, 10.1038/nrd3799. 22850786 PMC6493334

[34] E. Khanjani , A. Fergola , J. A. López Martínez , et al., “Capillary Microfluidics for Diagnostic Applications: Fundamentals, Mechanisms, and Capillarics,” Frontiers in Lab on a Chip Technologies 4 (2025): 1502127, 10.3389/frlct.2025.1502127.

[35] H. Fallahi , J. Zhang , H.‐P. Phan , and N.‐T. Nguyen , “Flexible Microfluidics: Fundamentals, Recent Developments, and Applications,” Micromachines 10, no. 12 (2019): 830, 10.3390/mi10120830. 31795397 PMC6953028

[36] J. E. Sosa‐Hernández , A. M. Villalba‐Rodríguez , K. D. Romero‐Castillo , et al., “Organs‐on‐a‐Chip Module: A Review from the Development and Applications Perspective,” Micromachines 9, no. 10 (2018): 536, 10.3390/mi9100536. 30424469 PMC6215144

[37] A. McMillan , G. Velvé‐Casquillas , E. Team , “Microreactors & Microfluidics in Chemistry–a Review,” Elveflow, https://elveflow.com/microfluidic‐reviews/microreactors‐and‐microfluidics‐in‐chemistry‐a‐review/.

[38] K. A. Gopinathan , A. Mishra , B. R. Mutlu , J. F. Edd , and M. Toner , “A Microfluidic Transistor for Automatic Control of Liquids,” Nature 622, no. 7984 (2023): 735–741, 10.1038/s41586-023-06517-3. 37880436 PMC10600001

[39] V. Iyer , Z. Yang , J. Ko , R. Weissleder , and D. Issadore , “Advancing Microfluidic Diagnostic Chips Into Clinical Use: A Review of Current Challenges and Opportunities,” Lab on a Chip 22, no. 17 (2022): 3110–3121, 10.1039/d2lc00024e. 35674283 PMC9798730

[40] M. I. Hajam and M. M. M. Khan , “Microfluidics: A Concise Review of the History, Principles, Design, Applications, and Future Outlook,” Biomaterials Science 12, no. 2 (2024): 218–251, 10.1039/D3BM01463K. 38108438

[41] Y. Lin , X. Zhou , and W. Cao , “3D‐Printed Hydraulic Fluidic Logic Circuitry for Soft Robots,” arXiv (2024), 10.48550/arXiv.2401.16827.

[42] A. Adamatzky , “A Brief History of Liquid Computers,” Philosophical Transactions of the Royal Society B: Biological Sciences 374, no. 1774 (1774): 20180372, 10.1098/rstb.2018.0372. PMC6553589 31006363

[43] W. Storr , “Universal Logic Gates and Complete Sets,” Basic Electronics Tutorials, https://www.electronics‐tutorials.ws/logic/universal‐gates.html (accessed 2025 October).

[44] S. Ilyas , A. Arevalo , E. Bayes , I. G. Foulds , and M. I. Younis , “Torsion Based Universal MEMS Logic Device,” Sensors and Actuators A: Physical 236 (2015): 150–158, 10.1016/j.sna.2015.10.039.

[45] R. Teja , “Introduction to Logic Gates,” ElectronicsHub, https://www.electronicshub.org/introduction‐to‐logic‐gates/ (accessed 2025 February).

[46] J. H. Sung and M. L. Shuler , “Prevention of Air Bubble Formation in a Microfluidic Perfusion Cell Culture System Using a Microscale Bubble Trap,” Biomedical Microdevices 11, no. 4 (2009): 731–738, 10.1007/s10544-009-9286-8. 19212816

[47] C. Lochovsky , S. Yasotharan , and A. Günther , “Bubbles No More: In‐Plane Trapping and Removal of Bubbles in Microfluidic Devices,” Lab on A Chip 12, no. 3 (2012): 595–601, 10.1039/c1lc20817a. 22159026

[48] M. Yang , N. Sun , Y. Luo , X. Lai , P. Li , and Z. Zhang , “Emergence of Debubblers in Microfluidics: A Critical Review,” Biomicrofluidics 16, no. 3 (2022): 031503, 10.1063/5.0088551. 35757146 PMC9217167

