# Supplementary material for: Responsive Open Microfluidic Logic for Mechanical Computing
Source: Adv Sci (Weinh). 2026 Sep 1;13(57):e77546. doi: 10.1002/advs.77546 (PMC13534792; doi:10.1002/advs.77546)
Supplement: Supplementary file 1 [file ADVS-13-e77546-s005.docx]

Supporting Information

Responsive Open Microfluidic Logic for Mechanical Computing

Roi Almog, Bat-El Pinchasik*

R. Almog, B.-E. Pinchasik

Tel-Aviv University

School of Mechanical Engineering

Faculty of Engineering

6997801

Tel-Aviv, Israel

R. Almog, B.-E. Pinchasik

Center for Physics and Chemistry of Living Systems, Tel Aviv University, Tel Aviv 69978, Israel

E-mail: [pinchasik@tauex.tau.ac.il](mailto:pinchasik@tauex.tau.ac.il)

Keywords: Flexural deformations, open Microfluidics, liquid diodes, logic gates, capillarity

Figure S1 shows the compressive forces and corresponding displacements used to apply mechanical inputs to the soft capillary circuits.

**Figure S1. Force as a function of normalized displacement for the liquid switch and liquid diode.** A) Force-normalized displacement curves measured during compression. The red curve corresponds to the liquid switch, and the blue curve corresponds to the liquid diode. B,C) Still-image sequences of the compression tests for the liquid switch (B) and liquid diode (C). Numbers indicate the image sequence. The yellow lines indicate the displacement corresponding to the compression distance of each component, and the red arrows indicate the images at maximum compression.

The operating mechanism of the liquid switch is illustrated in Figure S2. In the open state, a gap separates the two ends of the capillary pathway, preventing the liquid from reaching the downstream channel (Fig. S2A). In the closed state, the movable rectangular block is brought into contact with the channel, establishing a continuous wettable pathway between the two channel sections (Fig. S2B,I–II). The liquid then wets the surface of the block, propagates around it, and reaches the downstream channel, thereby restoring capillary connectivity across the switch (Fig. S2B,III–IV).

This mechanism explains the switching behavior shown in Figure 3. When the movable block is displaced away from the channel, the two sections remain separated and liquid propagation is blocked (Fig. 3B). When the block is positioned in contact with the channel, its wetted surface provides the capillary connection required for continued liquid propagation (Fig. 3A,C). Unlike the liquid diode, the switching mechanism does not rely on the formation of a liquid protrusion. Instead, the surface of the rigid movable block provides the continuous capillary pathway.

**Figure S2. Operating mechanism of the liquid switch.** A) Disconnected mode. I) Top-view image of the switch, with the dashed square indicating the region shown in the side view. II) Side-view cross-section showing the gap between the two ends of the capillary channel. (B) Connected mode. I,II) The movable rectangular block is in contact with the two ends of the channels, initiating liquid flow around the block. III,IV) The liquid wets the block and crosses the unit, allowing propagation into the downstream channel. Blue arrows indicate the direction of flow.

Still images from Video S6 for the half-adder operation with the input state X = 0 and Y=1 are shown in Figure S3. The half-adder operation starts from two initially unactuated gates: an AND gate forming the *Carry* circuit and an XOR gate forming the *Sum* circuit (Fig. S3A). The *Carry* and *Sum* circuits can operate in parallel, however, in this example, we first describe the *Carry* circuit. Since each circuit contains a single gate with two inputs, the inputs are denoted as X and Y, corresponding to compression along the x- and y-axes, respectively.

For the *Carry* circuit, compression is applied along the y-axis, corresponding to Y = 1 (Fig. S3B). The liquid propagates through the first diode but stops at the second diode in the same branch, which is oriented along the x-direction. Since X = 0, no compression is applied along the x-axis, the second diode remains blocking, and the liquid does not reach the star reservoir. Therefore, the *Carry* output is *Carry* = 0 (Fig. S3C).

For the *Sum* circuit, the same input condition is applied to the XOR gate, with compression along the y-axis (Fig. S3D). The liquid propagates through the first diode. In the parallel branch, the switch breaks the capillary bridge, preventing propagation through that branch. In the active branch, the switch remains connected and the capillary bridge is preserved. Therefore, the *Sum* output is *Sum* = 1 (Fig. S3E). In the Final result, for the input state X = 0 and Y = 1, the half-adder produces *Sum* = 1 and *Carry* = 0, corresponding to the binary addition 0 + 1 = 01.

**Figure S3. Half-adder operation for the input combination X=0 and Y=1.** Image series of the operation of a half-adder circuit captured from the Video S6. Images A-E indicate chronological order of the snapshots. Each circuit has its own logic diagram, consisting of one gate each. Red arrows indicate the direction of applied compression, and adjacent red text indicates the corresponding input (Y=1). The final readout is marked by a red dashed circle.

Correspondingly, still images from Video S6 for the half-adder operation with the input state X = 1 and Y = 1 are shown in Figure S4. Here, compression along the y-axis corresponds to Y = 1, and compression along the x-axis corresponds to X = 1. When both inputs are high (X=1, Y=1), the half-adder outputs *Sum* = 0 and *Carry* = 1, as expected for the binary addition 1+1=10.

**Figure S4. Figure S4.** Half-adder operation for the input combination X=1 and Y=1. Sequential still images illustrate the operation of the half-adder circuit. Panels A-H indicate the chronological sequence of liquid propagation. Each panel includes the corresponding logic diagram of the gate. Red arrows indicate the direction of the applied compression, while red labels denote the corresponding input. The final output is highlighted by a red dashed circle.

Similarly, we introduce image series of the Full Adder operation (Figs. S5-S7) for the input combinations A = 1, B = 1, and C_in_ = 1. The full-adder is divided into two separate physical circuits: a *Sum* circuit, composed of two cascaded XOR gates, and a *Carry* circuit, composed of two AND gates and two XOR gates (Fig. S5A). For each circuit, the corresponding logic tree is shown, and the gates are labeled according to their order of operation. In the *Sum* circuit, S1 denotes the first XOR gate, and S2 denotes the second and final XOR gate. In the *Carry* circuit, the gates are labeled C1–C4 according to their sequence in the logic tree (Fig. S5A).

**Figure S5. Full-adder *Sum* circuit for the input state A = 1, B = 1, and C_in_ = 1.** A) Complete full-adder circuit captured from the Video S7. The dashed blue box marks the *Sum* circuit and its logic tree. B) Sequential still images showing the operation of the *Sum* circuit. The numerical labels indicate the order of the snapshots. S1 denotes the first XOR gate and S2 denotes the second XOR gate, corresponding to the labels in the logic diagram. Dark red arrows indicate the direction of applied compression, and dark red text indicates the corresponding input value and input label. The final readout is marked by a red dashed circle.

Figure S6 focuses on the *Carry* circuit. In Fig. S6A, this circuit is marked by a dashed blue outline, and the operation of its first two gates is shown in Fig. S6B.

**Figure S6. Full-adder *Carry* circuit for the input state A = 1, B = 1, and C_in_ = 1:** operation of the first two gates, C1 and C2. A1) Complete full-adder circuit captured from Video S7. The dashed blue box marks the *Carry* circuit and its corresponding logic tree. A2) Enlarged view of the *Carry* logic tree. B) Sequential still images showing the operation of the *Carry* circuit. The numerical labels indicate the order of the snapshots. C1-C4 denote the gates according to their order in the *Carry* circuit: C1 is an AND gate, C2 is an XOR gate, C3 is an AND gate, and C4 is an XOR gate, corresponding to the labels in the logic diagram. Red arrows indicate the direction of applied compression, and red text indicates the corresponding input value and input label.

The operation of the last two gates of the *Carry* circuit is shown in Fig. S7B.

**Figure S7. Full-adder *Carry* circuit for the input state A = 1, B = 1, and C_in_ = 1: operation of the last two gates, C3 and C4.** A) Complete full-adder circuit captured from Video S7. The dashed blue box marks the *Carry* circuit and its corresponding logic tree. B) Sequential still images showing the operation of the *Carry* circuit. The numerical labels indicate the order of the snapshots. C1-C4 denote the gates according to their order in the *Carry* circuit: C1 is an AND gate, C2 is an XOR gate, C3 is an AND gate, and C4 is an XOR gate, corresponding to the labels in the logic diagram. Red arrows indicate the direction of applied compression, and Red text indicates the corresponding input value and input label. The final readout is marked by a red dashed circle.

Together with the previously obtained *Sum* output, the full-adder produces *Sum*=1 and *Carry* = 1 for the input combination A=1, B=1, and C_in_=1, corresponding to the binary addition 1+1+1=11 (decimal 3).

**Figure S8. circuit schematic of the dual-axis stepper motor control system.** A) An oblique view of the motorized mechanical actuation system. B) Circuit schematic of the control system. An Arduino UNO microcontroller controls two independently driven stepper motors through CNC-shield stepper drivers. Two pushbuttons provide manual triggering of the X and Y compression sequences, while a I2C LCD 1602 displays the system status. The actuation system is powered by an external 12 V DC supply.

Recent advances in structural capillarity have established open capillary microfluidics as a platform for passive and programmable liquid manipulation. While most existing systems rely on predefined geometry and surface properties, the present work introduces mechanically responsive capillary elements in which compression modulates liquid propagation and enables mechano-capillary logic. Table S1 compares representative microfluidic flow-control and logic approaches in terms of channel configuration, driving mechanism, logic implementation, environmental interaction, and main trade-offs.

**Table S1. Comparison of microfluidic flow-control and logic platforms.** Channel configuration, driving mechanism, logic implementation, interaction with the environment, and associated trade-offs.

| ***System*** | ***Microchannels*** | ***Driving mechanism*** | ***Logic implementation*** | ***Interaction with environment*** | ***Trade-off*** |
| --- | --- | --- | --- | --- | --- |
| *Closed, pump driven microfluidics*[^14,15,29-31,38^](https://www.zotero.org/google-docs/?YZJqwl) | *Closed* | *External pressure (pump)* | *External pump driven timing and synchronisation of flow throughout the circuit* | *Closed system - no direct interaction* | *1. Dependence on external pumps* |
|  |  |  |  |  | *2. Energy consumption* |
|  |  |  |  |  | *3. Channel clogging* |
|  |  |  |  |  | *4. No ability to interact with or intervene in the system during operation* |
| *Closed, fixed capillary microfluidics circuits*[^1-4^](https://www.zotero.org/google-docs/?YZJqwl) | *Closed* | *Spontaneous capillary flow (Negative Laplace pressure at the meniscus)* | *Surface design and liquid solid interaction* | *Closed system - no direct interaction* | *1. Relatively slow* |
|  |  |  |  |  | *2. Channel clogging* |
|  |  |  |  |  | *3. No ability to interact with or intervene in the system during operation* |
| *Open, fixed capillary microfluidics circuits*[^16,18,23^](https://www.zotero.org/google-docs/?YZJqwl) | *Open* | *Spontaneous capillary flow (Negative Laplace pressure at the meniscus)* | *Prefixed surface design and liquid solid interaction* | *Open system - direct interaction* | *1. Evaporation* |
|  |  |  |  |  | *2. Contamination from the environment* |
|  |  |  |  |  | *3. Relatively slow* |
|  |  |  |  |  | *4. Dependence liquid surface tension and wetting properties* |
| *Mechanically responsive open microfluidics in flexible capillary circuits* | *Open* | *Spontaneous capillary flow (Negative Laplace pressure at the meniscus)* | *Mechanical input* | *Open system - direct interaction* | *1. Evaporation* |
|  |  |  |  |  | *2. Contamination from the environment* |
|  |  |  |  |  | *3. Relatively slow* |
|  |  |  |  |  | *4. Dependence liquid surface tension and wetting properties* |

Videos **S1**-**S8** show the operations of open microfluidic basic units, logic gates, and the resulting two circuits: half-adder and full-adder, used to realize computational capabilities in open microfluidic systems. In addition, two applications of the open microfluidic circuits, including triggered reaction Videos **S9** and automated system Videos **S10**.

**Video S1. Zener diode.** Operation with and without mechanical compression.

**Video S2. liquid switch.** Operation with and without mechanical compression.

**Video S3. OR gate**

**Video S4. AND gate**

**Video S5. NOT gate**

**Video S6. XOR gate**

**Video S7. Half-adder circuit**

**Video S8. Full-adder circuit**

**Video S9. Controlled reaction of acetic acid and citric acid with sodium bicarbonate in an XOR gate**

**Video S10. Automated compression system: XOR gate**
